# Hybrid laparoscopic versus fully robot-assisted minimally invasive esophagectomy: an international propensity-score matched analysis of perioperative outcome

**DOI:** 10.1007/s00464-023-09911-0

**Published:** 2023-02-17

**Authors:** Jin-On Jung, Eline M. de Groot, B. Feike Kingma, Benjamin Babic, Jelle P. Ruurda, Peter P. Grimminger, Jens P. Hölzen, Yin-Kai Chao, Jan W. Haveman, Marc J. van Det, Philippe Rouanet, Frank Benedix, Hecheng Li, Inderpal Sarkaria, Mark I. van Berge Henegouwen, Gijs I. van Boxel, Philip Chiu, Jan-Hendrik Egberts, Rubens Sallum, Arul Immanuel, Paul Turner, Donald E. Low, Michal Hubka, Daniel Perez, Paolo Strignano, Matthias Biebl, M. Asif Chaudry, Christiane J. Bruns, Richard van Hillegersberg, Hans F. Fuchs, UGIRA Study Group

**Affiliations:** 1 Department of General, Visceral and Tumor Surgery, University Hospital Cologne, Kerpener Strasse 62, 50937 Cologne, Germany; 2 Department of Surgery, University Medical Center Utrecht, Utrecht, The Netherlands; 3 Department of General, Visceral and Transplant Surgery, University Medical Center Mainz, Mainz, Germany; 4 Department of General, Visceral and Transplant Surgery, University Hospital Münster, Münster, Germany; 5 Department of Thoracic Surgery, Chang Gung Memorial Hospital-Linkou Taoyuan, Taoyuan, Taiwan; 6 Department of Surgery, University Medical Center Groningen, Groningen, The Netherlands; 7 Department of Surgery, ZGT Hospital Almelo, Almelo, The Netherlands; 8 Department of Surgery, Institut Régional du Cancer de Montpellier, Montpellier, France; 9 Department of Surgery, University Hospital Magdeburg, Magdeburg, Germany; 10 Department of Thoracic Surgery, Ruijin Hospital Shanghai, Shanghai, China; 11 Department of Cardiothoracic Surgery, University of Pittsburgh Medical Center, Pittsburgh, USA; 12 Department of Surgery, University Medical Center Amsterdam, Amsterdam, The Netherlands; 13 Department of General Surgery, Portsmouth Hospitals NHS Trust, Portsmouth, UK; 14 Department of Surgery at Prince of Wales Hospital, The Chinese University of Hong Kong, Hong Kong, China; 15 Department of Surgery, Israelitisches Krankenhaus Hamburg, Hamburg, Germany; 16 Department of Digestive Surgery, University of São Paulo, São Paulo, Brasil; 17 Department of Surgery, Royal Victoria Infirmary Newcastle Upon Tyne, Newcastle upon Tyne, UK; 18 Department of Oesophagogastric Surgery, Lancashire Teaching Hospitals NHS Trust, Preston, UK; 19 Department of Thoracic Surgery and Thoracic Oncology, Virginia Mason Medical Center Seattle, Seattle, USA; 20 Department of General, Visceral, and Thoracic Surgery, University Medical Center Hamburg, Hamburg, Germany; 21 Department of General Surgery, Citta’ della Salute e della Scienza Turin, Turin, Italy; 22 Department of Surgery, Charité-Universitätsmedizin Berlin, Berlin, Germany; 23 Department of Academic Surgery, The Royal Marsden NHS Foundation Trust London, London, UK

**Keywords:** Robot-assisted minimally invasive esophagectomy, RAMIE, Hybrid laparoscopic approach, Propensity-score matching, Perioperative outcome, Complications

## Abstract

**Background:**

Currently, little is known regarding the optimal technique for the abdominal phase of RAMIE. The aim of this study was to investigate the outcome of robot-assisted minimally invasive esophagectomy (RAMIE) in both the abdominal and thoracic phase (full RAMIE) compared to laparoscopy during the abdominal phase (hybrid laparoscopic RAMIE).

**Methods:**

This retrospective propensity-score matched analysis of the International Upper Gastrointestinal International Robotic Association (UGIRA) database included 807 RAMIE procedures with intrathoracic anastomosis between 2017 and 2021 from 23 centers.

**Results:**

After propensity-score matching, 296 hybrid laparoscopic RAMIE patients were compared to 296 full RAMIE patients. Both groups were equal regarding intraoperative blood loss (median 200 ml versus 197 ml, *p* = 0.6967), operational time (mean 430.3 min versus 417.7 min, *p* = 0.1032), conversion rate during abdominal phase (2.4% versus 1.7%, *p* = 0.560), radical resection (R0) rate (95.6% versus 96.3%, *p* = 0.8526) and total lymph node yield (mean 30.4 versus 29.5, *p* = 0.3834). The hybrid laparoscopic RAMIE group showed higher rates of anastomotic leakage (28.0% versus 16.6%, *p* = 0.001) and Clavien Dindo grade 3a or higher (45.3% versus 26.0%, *p* < 0.001). The length of stay on intensive care unit (median 3 days versus 2 days, *p* = 0.0005) and in-hospital (median 15 days versus 12 days, *p* < 0.0001) were longer for the hybrid laparoscopic RAMIE group.

**Conclusions:**

Hybrid laparoscopic RAMIE and full RAMIE were oncologically equivalent with a potential decrease of postoperative complications and shorter (intensive care) stay after full RAMIE.

Oncological esophagectomy is a key component of curative treatment for resectable esophageal cancer and can be performed as a combination of open, laparoscopic/thoracoscopic and robot-assisted surgery [1–3]. Over the years, minimally invasive esophagectomy and especially robot-assisted minimally invasive esophagectomy (RAMIE) have gained in popularity, possibly allowing technical and postoperative advantages [4–8]. To date, research on the added value of a robotic system for esophagectomy has mainly focused on the thoracic phase while the added value of the robotic system during the abdominal phase has rarely been studied [9]. Most surgeons perform the abdominal phase laparoscopically or via laparotomy [10]. Moreover, the decision to be operated via a fully robotic or only partially robotic approach may depend on multiple patient characteristics such as body mass index and comorbidities. A recent review elaborated on the effects of robot assistance during the abdominal phase of RAMIE suggesting its non-inferiority compared to conventional laparoscopic abdominal approaches [11]. However, it remains unclear how the robot-assisted abdominal phase during RAMIE relates to laparoscopy regarding oncological safety and perioperative complications. Therefore, the aim of this study was to compare hybrid laparoscopic RAMIE to full RAMIE for patients with esophageal cancer in an international propensity-score matched cohort study.

## Materials and methods

Data were acquired from the prospectively maintained database from the Upper GI International Robotic Association (UGIRA) [12]. The UGIRA group was initiated in 2017 as a worldwide group investigating robotic surgery in upper gastrointestinal cancer and provides data on perioperative care of patients who underwent robotic esophagogastric surgery. Participating centers can provide data for the UGIRA database without a minimum number of robotic procedures. This design was intentionally chosen to also compare the first robot-assisted operations which might be under the influence of a learning curve. The registry consists of 23 participating centers and the UGIRA study group has a central institutional review board approval at the University Medical Center of Utrecht (17/837). For each participating center local ethical approval was either obtained or waived by the local ethical committee. The research proposal was reviewed by the scientific committee of UGIRA and was approved. This paper follows to the STROBE guidelines for observational cohort studies [13].

### Patients, procedures and tumor entity

All patients of the UGIRA group who underwent full RAMIE and hybrid laparoscopic RAMIE for esophageal cancer between 2017 and 2021 were included. Full RAMIE consists of both a robot-assisted abdominal phase and a robot-assisted thoracic phase while the hybrid laparoscopic group consists of a laparoscopic abdominal phase and a robot-assisted thoracic phase. In this study, only procedures with curative intention and intrathoracic anastomosis (Ivor-Lewis) were included. One essential inclusion criterion defined adenocarcinoma or squamous cell carcinoma as acceptable tumor entities based on the preoperative histology during primary diagnosis. However, final histopathology could eventually differentiate other tumor entities such as mixed adenoneuroendocrine carcinoma (MANEC), poorly differentiated carcinoma or small cell carcinoma as well (named as other tumor entities).

### Outcomes

The primary endpoint was postoperative complications according to Clavien Dindo grade 3a or higher. Secondary endpoints were intraoperative adverse events, in-hospital mortality, postoperative complications, and oncological outcomes including radical resection (R0) rates and lymph node yield. The eighth TNM edition was used for both clinical and pathological TNM stage. The definition of the College of American Pathologists was used for radical resection (i.e., no tumor cells within the resection margins).

### Statistical analysis

**Fig. 1:**
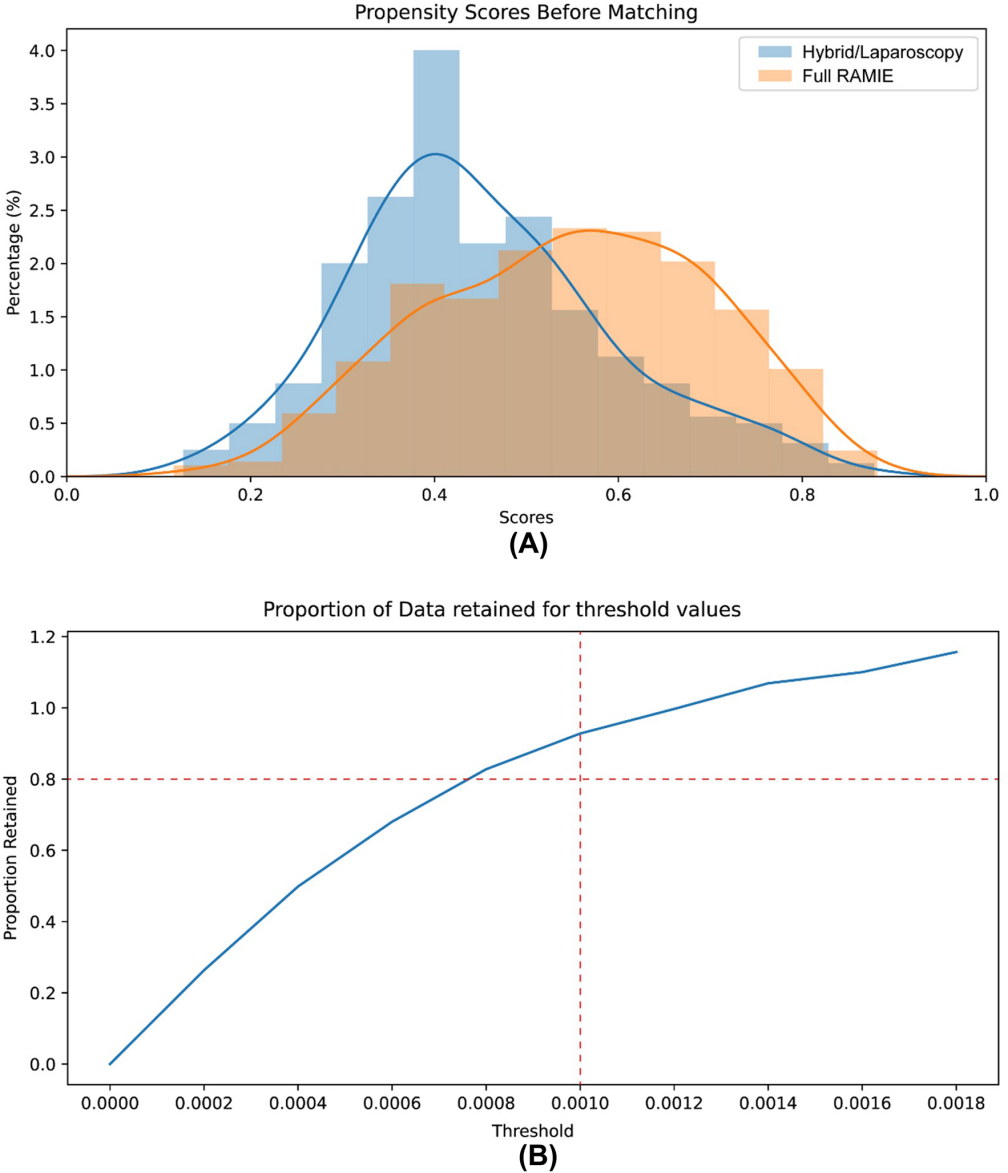
Overlap of data points with propensity-score plotted against data availability (see **A**). The augmentation of the PSM threshold does not necessarily lead to higher case numbers which is why a threshold of 0.001 was chosen with a retained proportion of > 80% (see **B**)

Data management, missingness imputation and propensity-score matching (PSM) were all realized via Python 3.9 [14] within the integrated development environment of Visual Studio Code (Version 1.59). Patients who underwent full RAMIE were compared to patients who underwent hybrid laparoscopic RAMIE. To account for missing data, case-specific and variable-specific missingness of more than 25% was excluded. Eventually, the overall rate of missing data in the whole dataset was calculated as 2.0% which is widely accepted as a legitimate threshold for imputation. We performed multiple imputations with *n* = 1000 iterations via Iterative Imputer from Sci–kit learn [15]. After multiple imputation, a propensity-score matching analysis was performed via the Python package *pymatch* (adapted from the R package Matching [16]) to reduce the effect of known confounders to a minimum. As potential confounders, all variables were utilized which were available before surgery and which were considered as potentially relevant for the decision to either belong to the full RAMIE or hybrid laparoscopic RAMIE group. Through logistic regression, a propensity-score was calculated for each patient based on the selected characteristics displayed in Table 1. Matched study groups were created using nearest-neighbor one-to-one matching without replacement. A threshold of 0.001 was calculated to prevent poor matches after optimizing the threshold and simultaneous maximization of retained proportion according to the overlap of both groups (demonstrated in Fig. 1). After matching, the further comparison between full RAMIE and hybrid laparoscopic RAMIE was performed using Chi^2^-square tests for binary data, Mann–Whitney U test for ordinal data and student’s t-test for continuous data. A *p*-value of less than 0.05 was considered as statistically significant. StataSE Version 15.0 (by StataCorp LLC, College Station, TX) was eventually used for final statistical analysis after matching.

**Table 1:** Preoperative variables used for propensity-score matching

Parameter	Before matching	*p*-value	After matching	*p*-value
1. Hybrid laparoscopic RAMIE 2. Full RAMIE	*n* = 319 *n* = 488		*n* = 296 *n* = 296	
Age at diagnosis - Hybrid laparoscopic RAMIE - Full RAMIE	Mean (95%-CI) 64.2 (63.2–65.2) 64.5 (63.6–65.4)	0.6308 (*t*-test)	Mean (95%-CI) 64.5 (63.5–65.5) 64.2 (63.1–65.3)	0.7593 (t-test)
Gender - Hybrid laparoscopic RAMIE - Full RAMIE	Female (%) 55 (17.2) 98 (20.1)	0.314 (*χ*^2^-test)	Female (%) 62 (20.9%) 51 (17.2%)	0.744 (*χ*^2^-test)
Body mass index (in kg/m^2^) - Hybrid laparoscopic RAMIE - Full RAMIE	Mean (95%-CI): 26.5 (26.0–27.0) 26.1 (25.7–26.5)	0.2942 (*t*-test)	Mean (95%-CI): 25.7 (25.2–26.1) 26.2 (25.7–26.7)	0.1213 (*t*-test)
ASA score - Hybrid laparoscopic RAMIE * - ASA 1* * - ASA 2* * - ASA 3* * - ASA 4* - Full RAMIE * - ASA 1* * - ASA 2* * - ASA 3* * - ASA 4*	*n* (%) 28 (8.8) 207 (64.9) 83 (26.0) 1 (0.3) 29 (5.9) 252 (51.6) 203 (41.6) 4 (0.8%)	** < 0.0001** (*U*-test)	*n* (%) 9 (3.0) 177 (59.8) 108 (36.5) 2 (0.7) 22 (7.4) 175 (59.1) 98 (33.1) 1 (0.3)	0.1155 (*U*-test)
cT-status - Hybrid laparoscopic RAMIE * - cT1a/b* * - cT2* * - cT3* * - cT4a/b* - Full RAMIE * - cT1a/b* * - cT2* * - cT3* * - cT4a/b*	*n* (%) 46 (9.4) 94 (19.3) 324 (66.4) 24 (4.9) 29 (9.1) 57 (17.9) 227 (71.2) 6 (1.9)	0.8984 (*U*-test)	*n* (%) 19 (6.4) 58 (19.6) 214 (72.3) 5 (1.7) 26 (8.8) 59 (19.9) 196 (66.2) 15 (5.1)	0.8383 (*U*-test)
cN-status - Hybrid laparoscopic RAMIE * - cN0* * - cN1* * - cN2* * - cN3* - Full RAMIE * - cN0* * - cN1* * - cN2* * - cN3*	*n* (%) 99 (31.0) 178 (55.8) 39 (12.2) 3 (0.9) 186 (38.1) 238 (48.8) 53 (10.9% 11 (2.3)	0.1026 (*U*-test)	*n* (%) 116 (39.2) 142 (48.0) 35 (11.8) 3 (1.0) 106 (35.8) 153 (51.7) 31 (10.5) 6 (2.0)	0.5870 (*U*-test)
cMX-status - Hybrid laparoscopic RAMIE - Full RAMIE	Yes (%) 8 (2.5) 9 (1.8)	0.521 (*χ*^2^-test)	Yes (%) 6 (2.0) 3 (1.0)	0.314 (*χ*^2^-test)

Parameter	Before matching	*P*-value	After matching	*P*-value
1. Hybrid laparoscopic RAMIE 2. Full RAMIE	*n* = 319 *n* = 488		*n* = 296 *n* = 296	
Year of esophagectomy - Hybrid laparoscopic RAMIE * - 2017* * - 2018* * - 2019* * - 2020* * - 2021* - Full RAMIE * - 2017* * - 2018* * - 2019* * - 2020* * - 2021*	*n* (%) 51 (16.0) 80 (25.1) 141 (44.2) 37 (11.6) 10 (3.1) 124 (25.4) 155 (31.8) 125 (25.6) 75 (15.4) 9 (1.8)	**0.0006** (*U*-test)	*n* (%) 69 (23.3) 69 (23.3) 124 (41.9) 28 (9.5) 6 (2.0) 68 (23.0) 97 (32.8) 84 (28.4) 43 (14.5) 4 (1.4)	0.3264 (*U*-test)
Neoadjuvant therapy - Hybrid laparoscopic RAMIE * - No neoadjuvant therapy* * - Radiochemotherapy* * - Chemotherapy alone* * - Other* - Full RAMIE * - No neoadjuvant therapy* * - Radiochemotherapy* * - Chemotherapy alone* * - Other*	*n* (%) 35 (11.0) 220 (69.0) 60 (18.8) 4 (1.25) 110 (22.5) 255 (52.3) 115 (23.6) 8 (1.64)	0.2049 (*U*-test)	*n* (%) 24 (8.1) 219 (74.0) 49 (16.6) 4 (1.4) 61 (20.6) 158 (53.4) 74 (25.0) 3 (1.0)	0.4625 (*U*-test)
Cardiological comorbidities - Hybrid laparoscopic RAMIE - Full RAMIE	Yes (%) 94 (26.8) 131 (29.5)	0.417 (*χ*^2^-test)	Yes (%) 84 (28.4) 77 (26.0)	0.518 (*χ*^2^-test)
Diabetes - Hybrid laparoscopic RAMIE - Full RAMIE	Yes (%) 42 (13.2) 71 (14.5)	0.580 (*χ*^2^-test)	Yes (%) 45 (15.2) 45 (15.2)	1.000 (*χ*^2^-test)
Neurological comorbidities - Hybrid laparoscopic RAMIE - Full RAMIE	Yes (%) 23 (7.2) 29 (5.9)	0.473 (*χ*^2^-test)	Yes (%) 17 (5.7) 18 (6.1)	0.862 (*χ*^2^-test)
History of malignant disease - Hybrid laparoscopic RAMIE - Full RAMIE	Yes (%) 16 (5.0) 46 (9.4)	**0.021** (*χ*^2^-test)	Yes (%) 23 (7.8) 23 (7.8)	1.000 (*χ*^2^-test)
Pulmonary comorbidities - Hybrid laparoscopic RAMIE - Full RAMIE	Yes (%) 43 (13.5) 79 (16.2)	0.294 (*χ*^2^-test)	Yes (%) 46 (15.5) 45 (15.2)	0.909 (*χ*^2^-test)
Vascular comorbidities - Hybrid laparoscopic RAMIE - Full RAMIE	Yes (%) 93 (29.2) 167 (34.2)	0.132 (*χ*^2^-test)	Yes (%) 96 (32.4) 93 (31.4)	0.791 (*χ*^2^-test)
Other comorbidities - Hybrid laparoscopic RAMIE - Full RAMIE	Yes (%) 77 (24.1) 143 (29.3)	0.107 (*χ*^2^-test)	Yes (%) 89 (30.1) 87 (29.4)	0.857 (*χ*^2^-test)

Significant values (*p* < 0.05) are given in bold

*RAMIE* robot-assisted minimally invasive esophagectomy, 95%-CI = 95% confidence interval, *ASA* American society of anesthesiologists

## Results

### Study population

A total of 807 patients underwent Ivor-Lewis esophagectomy and were included for propensity-score matching. Table 1 summarizes all preoperative variables which were used for logistic regression to achieve propensity-score matching. Several parameters such as ASA score (*p* < 0.0001), year of esophagectomy (*p* = 0.0002) and history of malignant disease (*p* = 0.021) were significantly different between groups before matching. Figure 1A shows the overlap of patients in both groups (full RAMIE versus hybrid laparoscopic RAMIE) with their propensity-score plotted on the x-axis. To maximize data similarity, propensity-score matching was eventually performed with an average accuracy of the score of 65.11% based on the selected preoperative variables. In Fig. 1B, the threshold is depicted in relation to the retained proportion of cases. Finally, 296 patients were matched for each group. The last two columns of Table 1 demonstrate the frequency distributions and test statistics of both matched groups with all parameters not showing any significant differences.

### Hybrid laparoscopic RAMIE versus full RAMIE

**Fig. 2:**
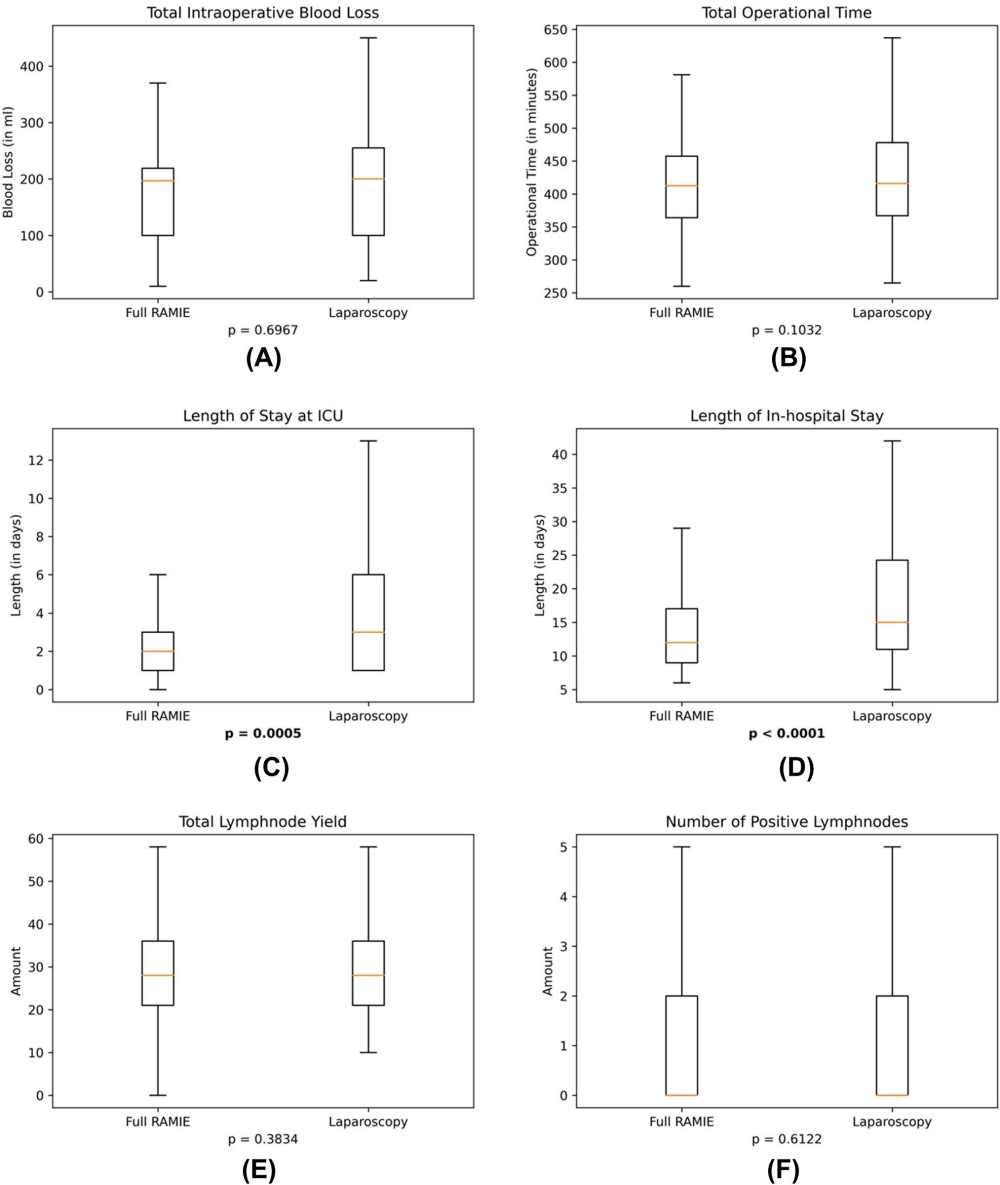
Box plots for continuous outcome parameters: Significant differences were found for length of stay on ICU (see **C**, *p* = 0.0005) and for total length of in-hospital stay (see **D**, *p* < 0.0001). Box plots for **A** total intraoperative blood loss, **B** total operational time, **C** length of stay on ICU, **D** length of in-hospital stay, **E** total lymphnode yield, **F** number of positive lymphnodes. *ICU* intensive care unit

Table 2 demonstrates all outcome variables and test statistics for both full RAMIE and hybrid laparoscopic RAMIE. Figure 2 shows all continuous outcome variables represented as box plots.

**Table 2:** Intraoperative and postoperative outcome variables with according test statistics after propensity-score matching of both groups hybrid laparoscopic RAMIE and full RAMIE

Parameter	Hybrid laparoscopic RAMIE (*n* = 296)	Full RAMIE (*n* = 296)	*p*-value
Intraoperative blood loss (in ml)	Median (IQR): 200.0 (100.0–260.0)	Median (IQR): 197.0 (100.0–219.0)	0.6967 (*t*-test)
Operational time (in minutes)	Mean (95%-CI): 430.3 (420.7–439.9)	Mean (95%-CI): 417.7 (406.0–429.4)	0.1032 (*t*-test)
Surgical technique - Circular stapler - Linear stapler - Hand-sewn	*n* (%) 139 (47.0) 64 (21.6) 93 (31.4)	*n* (%) 173 (58.5) 26 (8.8) 97 (32.8)	0.0953 (*U*-test)
Anastomosis type - End-to-side - End-to-end - Side-to-side	*n* (%) 217 (73.3) 12 (4.1) 67 (22.6)	*n* (%) 212 (71.6) 57 (19.3) 27 (9.1)	0.5366 (*U*-test)
Conversion to open surgery During abdominal phase	Yes (%): 7 (2.4)	Yes (%): 5 (1.7)	0.560 (*χ*^2^-test)
Conversion to open surgery during thoracic phase	Yes (%): 4 (1.4)	Yes (%): 8 (2.7)	0.243 (*χ*^2^-test)
Length of stay ICU (in days)	Median (IQR): 3 (1–6)	Median (IQR): 2 (1–3)	**0.0005** (*t*-test)
Length of in-hospital stay (in days)	Median (IQR): 15 (11–25)	Median (IQR): 12 (9–17)	** < 0.0001** (*t*-test)
Tumor histology - Adenocarcinoma - Squamous cell carcinoma - Others	*n* (%) 250 (84.5) 37 (12.5) 9 (3.0)	*n* (%) 225 (76) 65 (22.0) 6 (2.0)	**0.0145** (*U*-test)
Total lymph node yield	Mean (95%-CI): 30.4 (29.0–31.7)	Mean (95%-CI): 29.5 (28.0–30.9)	0.3834 (*t*-test)
Positive lymph nodes	Median (IQR): 0 (0–2)	Median (IQR): 0 (0–2)	0.6122 (*t*-test)
R1 status	Yes (%) 13 (4.4%)	Yes (%) 11 (3.7%)	0.677 (*χ*^2^-test)

Parameter	Hybrid laparoscopic RAMIE (*n* = 296)	Full RAMIE (*n* = 296)	*p*-value
Anastomotic leakage	Yes (%): 83 (28.0)	Yes (%): 49 (16.6)	**0.001** (*χ*^2^-test)
Clavien Dindo grade ≥ 3a	Yes (%): 134 (45.3)	Yes (%): 77 (26.0)	** < 0.001** (*χ*^2^-test)
Highest Clavien Dindo grade - Grade 0 - Grade 1 - Grade 2 - Grade 3a - Grade 3b - Grade 4 - Grade 5	*n* (%) 85 (28.7) 24 (8.11) 53 (17.9) 80 (27.0) 23 (7.8) 22 (7.4) 9 (3.0)	*n* (%) 141 (47.6) 24 (8.1) 54 (18.2) 36 (12.2) 23 (7.8) 10 (3.4) 8 (2.7)	** < 0.0001** (*U*-test)
Readmission to ICU	Yes (%): 41 (17.5)	Yes (%): 27 (11.2)	0.071 (*χ*^2^-test)
Readmission to hospital	Yes (%): 29 (9.8)	Yes (%): 38 (13.2)	0.241 (*χ*^2^-test)
Hospital-acquired pneumonia	Yes (%): 47 (15.9)	Yes (%): 49 (16.6)	0.824 (*χ*^2^-test)
Atrial fibrillation	Yes (%): 37 (12.5)	Yes (%): 30 (10.1)	0.364 (*χ*^2^-test)
Chyle leakage	Yes (%): 15 (5.1)	Yes (%): 17 (5.7)	0.716 (*χ*^2^-test)
In-hospital mortality	Yes (%) 9 (3.0)	Yes (%) 8 (2.7)	0.806 (*χ*^2^-test)

Significant values (*p* < 0.05) are given in bold

*ICU* intensive care unit, *IQR* interquartile range, 95%-CI 95% confidence interval

**Fig. 3:**
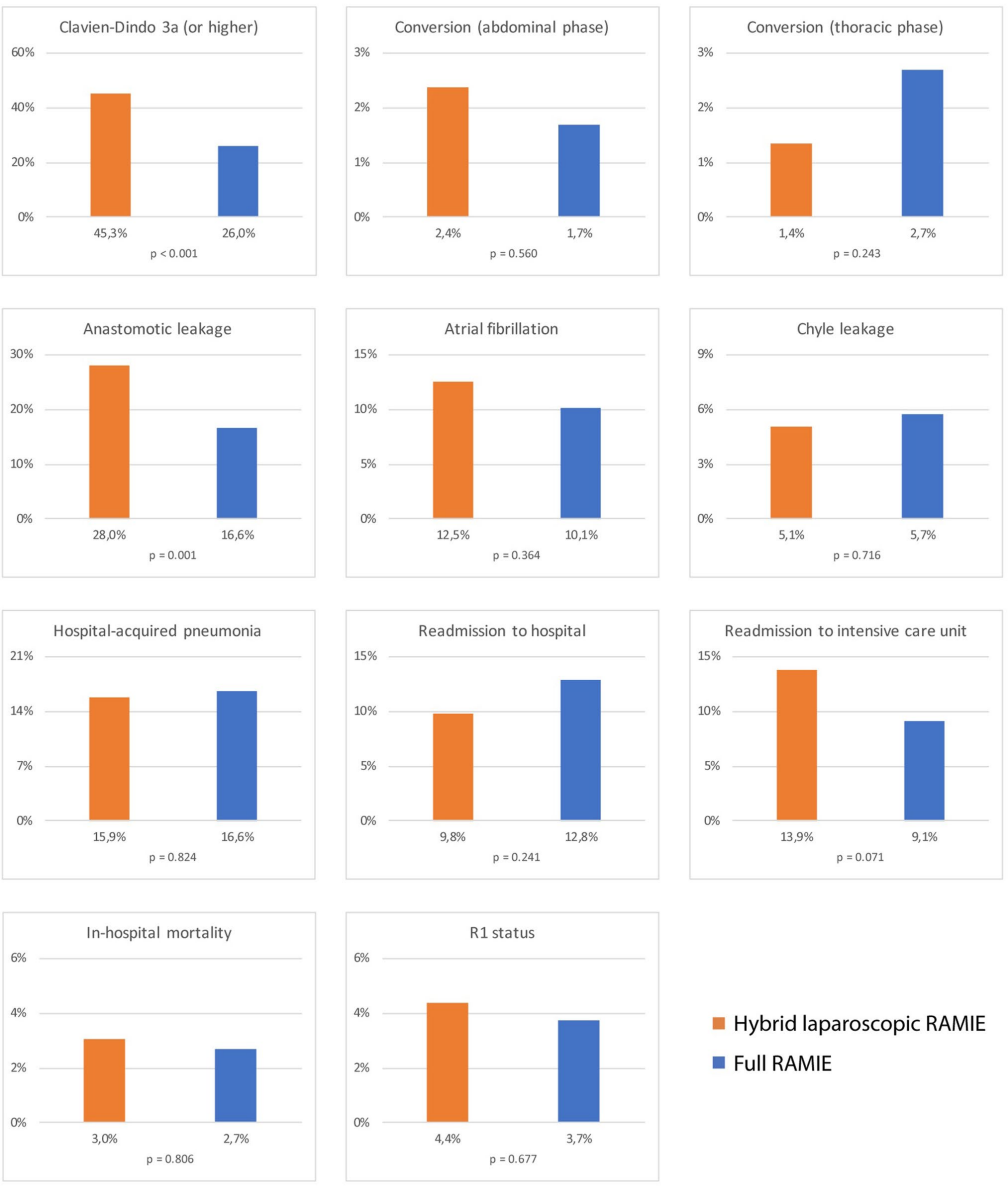
Bar graphs of binary outcome parameters: Significant differences were found for complications according to Clavien Dindo grade 3a or higher (*p* < 0.001) and anastomotic leakage (*p* = 0.001). *RAMIE* robot-assisted minimally invasive esophagectomy

Intraoperative parameters such as blood loss (*p* = 0.6967) and operational time (*p* = 0.1032) were not significantly different between both groups. Median intraoperative blood loss was measured as 200 ml for hybrid laparoscopic RAMIE and as 197 ml for full RAMIE. Mean operational time was averaged 430.3 min for hybrid laparoscopic RAMIE compared to 417.7 min for full RAMIE. Significant differences could be found for the length of stay (LOS) on intensive care unit (median LOS of 3 days for hybrid laparoscopic RAMIE versus 2 days for full RAMIE, *p* = 0.0005) and total in-hospital stay (median LOS of 15 days versus 12 days, *p* < 0.0001) (Fig. 3)

Oncological outcome parameters such as radical resection (R0) rates (95.6% for hybrid laparoscopic RAMIE versus 96.3% for full RAMIE, *p* = 0.8526) and total lymph node yield (mean 30.4 for hybrid laparoscopic RAMIE versus 29.5 for full RAMIE, *p* = 0.3834) were comparable between both groups. Likewise, the number of positive lymph nodes in the final histopathology did not differ between both groups (median 0 for both RAMIE groups, *p* = 0.6122). The conversion rate to open surgery during the abdominal phase was 2.4% in the hybrid laparoscopic RAMIE group compared to 1.7% in the full RAMIE group (*p* = 0.560). During the thoracic phase open surgery occurred in 1.4% of hybrid laparoscopic RAMIE cases and in 2.7% of full RAMIE cases (*p* = 0.243).

Postoperative complications with Clavien Dindo grade 3a or higher appeared more frequently in the hybrid laparoscopic RAMIE group (45.3% versus 26.0%, *p* < 0.001). This is confirmed via the *U*-test for the most severe Clavien Dindo grade reported for the individual patients (*p* < 0.0001). The overall postoperative complication rate was also higher in the hybrid laparoscopic RAMIE group (65.2% versus 48.3%, *p* < 0.001), including specific complications such as anastomotic leakage (28.0% versus 16.6%, *p* = 0.001). Readmission rates either to intensive care unit (17.5% for hybrid laparoscopic RAMIE versus 11.2% for full RAMIE, *p* = 0.071) or to hospital (9.8% versus 13.2%, *p* = 0.241) did not differ significantly between both groups. The rate of hospital-acquired pneumonia after surgery also did not differ between both groups (15.9% for hybrid laparoscopic RAMIE versus 16.6% for full RAMIE, *p* = 0.824).

## Discussion

This propensity-score matched analysis compared hybrid laparoscopic RAMIE to full RAMIE and suggests that full RAMIE may be superior in terms of overall postoperative complications according to Clavien Dindo grade 3a or higher. A significantly lower percentage of anastomotic leakage was observed after full RAMIE as opposed to the hybrid laparoscopic RAMIE group. In addition, the length of in-hospital stay after full RAMIE was significantly shorter than after hybrid laparoscopic RAMIE. Oncological outcomes (such as radical resection rates or lymph node yield) and intraoperative parameters including operation time were equal for both procedures.

To date, only few studies have focused specifically on the abdominal phase by comparing full RAMIE with hybrid laparoscopic RAMIE [17–20]. For instance, a retrospective multicenter study by Grimminger et al. compared 175 full RAMIE procedures to 67 hybrid (either laparoscopic or open laparotomy) RAMIE procedures and demonstrated that full RAMIE was associated with significantly lower postoperative complications including anastomotic leakage and respiratory failure [20]. Since there is not much evidence in the current literature, it is necessary to reflect on potential benefits of the robotic abdominal approach. Thus, shorter operation times after full RAMIE and a more precise dissection and reduction in surgical trauma of the gastric conduit could theoretically lead to less complications such as anastomotic leakage of the esophagogastrostomy [20–22]. On the other hand, financial expenses of a robotic system and its maintenance are often debated. It has been shown that hybrid laparoscopic RAMIE can be performed with comparable costs in comparison to full RAMIE in the setting of a high-volume European medical center [23]. If robotic assistance does truly lead to a decrease in postoperative complications, it is thinkable that costs could be saved on the long run regarding avoidable time and resources during intensive care and postoperative course [24].

Concerning the limitations of this study, it is to state that the retrospective design based on the UGIRA database may not respect standardized operational steps of the participating centers (such as the implementation of a feeding jejunostomy during the abdominal phase). Similarly, the acquisition of data regarding abdominal lymph node yield and operational time during the abdominal phase is heterogeneously available with a significant missingness due to different approaches by the centers. As another important limitation, it is necessary to discuss a potential learning curve effect leading to the concordant result that a robot-assisted abdominal phase might be superior to laparoscopy during RAMIE. It is very likely that a learning curve effect is involved in the hybrid laparoscopic RAMIE group. A robotic system is generally implemented in the thoracic phase at first place, and after completing the learning curve for the thoracic phase the robotic system may also be implemented for the abdominal phase. Hence, it may be possible that the full RAMIE cases included in this analysis were more frequently performed by a team that has more robotic experience. Consequently, it may be possible that the hybrid laparoscopic RAMIE group consists of procedures performed by surgeons who are undergoing the learning curve for RAMIE. According to the current literature, the learning curve for RAMIE is generally completed after 45–70 cases with the possibility of being shortened by following a structured training pathway that involves proctoring, and modular approaches may help to further reduce time to proficiency [25–28]. On the other hand, there is also a learning curve for the robot-assisted abdominal phase, although only few studies have dealt with this question and allegedly found a plateau phase after 14–22 cases [29, 30]. Moreover, the learning curve for non-robotic total MIE has also been reported to be relatively high with 119 cases [31]. The effect of the learning curve may be significant for the results of the presented study since the UGIRA registry holds data from centers that are yet in their learning curve. Anyhow, in order to solely compare the robot-assisted abdominal phase to laparoscopy a cohort without a learning curve effect is needed. In this way, only cases after completion of the learning curve for both the thoracic as well as the abdominal phase could be included in a follow-up study. Finally, the significance of a learning curve effect during the abdominal phase has to be elucidated especially in this setting of two cavity surgery where the thoracic phase is performed robotically in any case.

A strength of this study is the fact that it includes a large and international multicenter cohort representing the real practice of specialized hospitals. The UGIRA study group offers the unique opportunity to conduct comparative studies based on standardized procedures and a rigorous selection of participating medical centers. This study also features a strong methodology with a state-of-the-art statistical implementation for data handling, missingness imputation and propensity-score matching.

The current study showed that the use of a robotic system in the abdominal phase during RAMIE achieves comparably good postoperative outcomes. The study suggests that the implementation of a robotic system during the abdominal phase is safe without compromising histopathological results. In the future, it is inevitable to perform prospective and randomized studies investigating whether full RAMIE is truly superior to hybrid laparoscopic RAMIE regarding complications and long-term expenses.
